# Cadaveric Case Report of Pelvic and Abdominal Anatomy in an Individual With a Müllerian Duct Anomaly

**DOI:** 10.7759/cureus.80729

**Published:** 2025-03-17

**Authors:** Fiona Lane, Alexander Jonson, Kevin Hare, Megan Forgie, Michelle Riedel, Anna Hardin

**Affiliations:** 1 Obstetrics and Gynecology, Western University of Health Sciences College of Osteopathic Medicine of the Pacific-Northwest, Lebanon, USA; 2 Emergency Medicine, Western University of Health Sciences, Lebanon, USA; 3 Internal Medicine, Western University of Health Sciences College of Osteopathic Medicine of the Pacific-Northwest, Lebanon, USA; 4 Anatomical Sciences, Western University of Health Sciences College of Osteopathic Medicine of the Pacific-Northwest, Lebanon, USA

**Keywords:** cadaveric dissection, cervical agenesis, leiomyomas, mayer-rokitansky-küster-hauser (mrkh) syndrome, mullerian duct anomaly

## Abstract

Müllerian duct anomalies (MDAs) are a congenital abnormality of the paramesonephric ducts that result in malformation of the uterus, cervix, and/or vagina. Here, the authors present a class I MDA potentially suggesting Mayer-Rokitansky-Küster-Hauser (MRKH) syndrome with two masses incidentally found upon dissection into the uterine cavity. Only a single MRKH syndrome with uterine masses in cadavers has been previously described in the literature. The authors aim to enrich the body of gynecological and reproductive research currently available by increasing understanding of the types of MDAs and to increase anatomical understanding of the condition. This will help anatomists and students of anatomy identify MDAs during dissection, which will ultimately help physicians more appropriately counsel future patients in reproductive health and direct them to appropriate treatment.

## Introduction

Embryological development of the uterus, cervix, and superior vagina requires differentiation and fusion of the paramesonephric (Müllerian) ducts [1]. Anomalous development of the paramesonephric ducts can produce variable uterine anatomies, including septate, bicornuate, or unicornuate uterus, as well as hypoplasia or agenesis of all or part of the uterus and/or cervix [2]. These conditions, typically called Müllerian duct anomalies (MDAs), are classified into nine categories by the American Society for Reproductive Medicine (ASRM) [3]. MDAs are rare, affecting 5.5% of a random, anatomically female population based on a meta-analysis of 94 studies [4].

Mayer-Rokitansky-Küster-Hauser (MRKH) syndrome is a rare congenital anomaly characterized by agenesis of paramesonephric duct structures in patients with normal secondary sexual characteristics and 46XX karyotype [5]. Occurring in one in 4,000-5,000 live female births, MRKH syndrome is the most common clinical syndrome of paramesonephric duct dysgenesis and the second most common cause of primary amenorrhea after gonadal dysgenesis [6]. The syndrome is split into two subtypes based on the presence (type B or “atypical” MRKH) or absence (type A or “typical” MRKH) of extragenital involvement, including renal, skeletal, hearing, cardiac, and other anomalies [7]. Although the etiology of this syndrome is not completely understood, multiple genes of the HOXA group have been indicated due to their role in the normal embryological development of the paramesonephric ducts [6].

Patients with MRKH syndrome are typically diagnosed with uterine agenesis in adolescence after consulting medical attention for amenorrhea, inability to have intercourse, and/or dyspareunia. Cyclic abdominal pain and signs of endometriosis may occur in those with rudimentary horns of the uterus and functional endometrium, but most patients with MRKH syndrome have normal female endocrine function and normally functioning fallopian tubes and ovaries [6]. Rarely in the medical literature have patients with MRKH syndrome presented with multiple leiomyomas [8,9]. The authors were able to find a single published cadaveric study of the pelvic and abdominal anatomy of individuals with MRKH syndrome or uterine and cervical hypoplasia published during the composition of this case report [10].

Cadaveric study of anatomical variation allows the observation of specific aspects of organ size, position, and morphology, as well as neurovascular variants, that may not be visible with medical imaging. The authors present here a cadaveric study of an individual with potential Müllerian duct dysgenesis to serve as an example of the variation among Müllerian anomalies that can make their classification and diagnosis challenging. 

This article was previously presented as a poster presentation at the Western University of the Health Sciences Research Symposium on December 13, 2022.

## Case presentation

The donor cadaver was obtained as part of the Western University of Health Sciences Willed Body Program. During first-year medical students’ dissection of the pelvic cavity of a 78-year-old anatomically female donor, two masses were observed inferior to the broad ligament after layer dissection of the abdomen was performed. These were initially thought to be the horns of a bicornuate uterus, and detailed dissection, including pelvic hemisection, was performed to fully describe the anatomy of the pelvis and abdomen.

Renal

**Figure 1:**
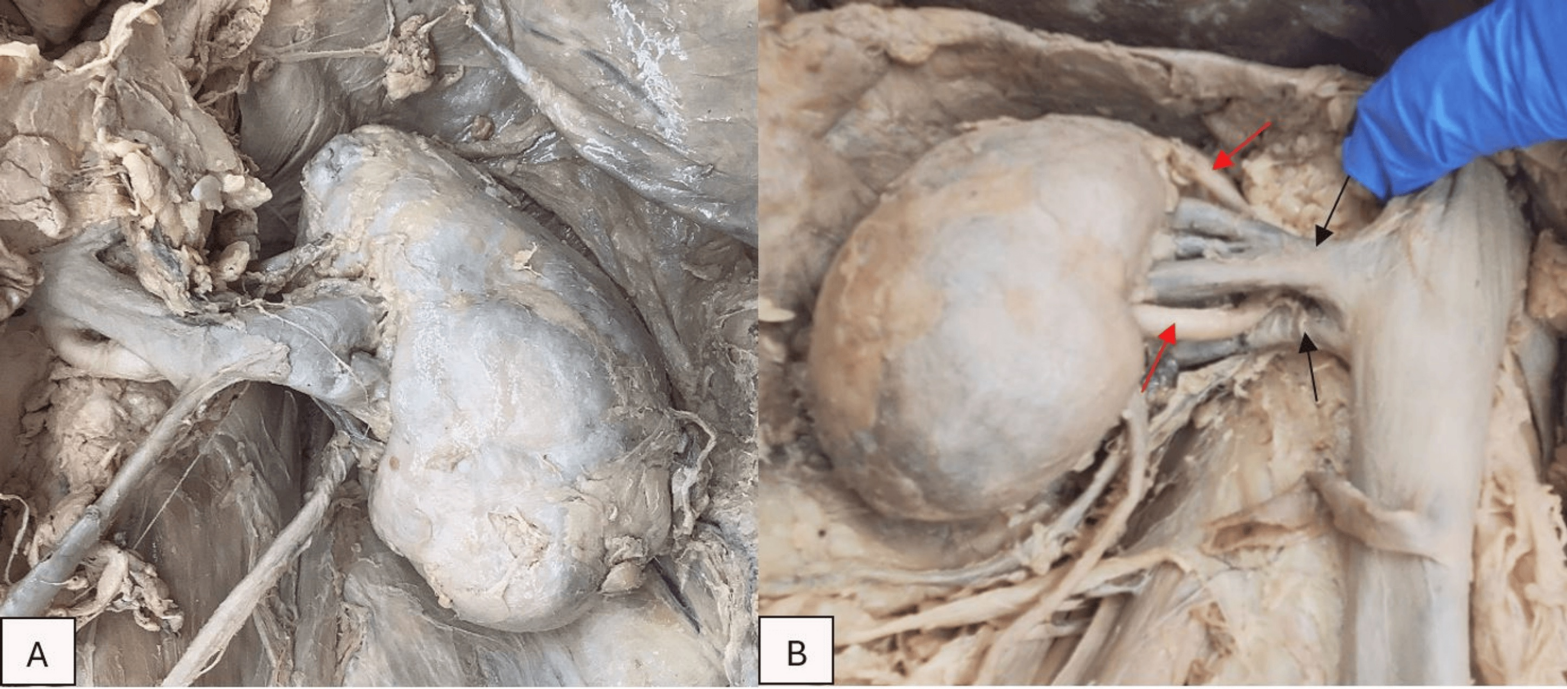
A) Left kidney with an irregular shape, B) right kidney with two renal arteries (red arrows) and two renal veins (black arrows). The renal arteries were full dissected back to the descending aorta (not seen in the image).

The right and left kidneys measured 100.95 mm and 99.57 mm from pole to pole, respectively. All measurements were obtained utilizing spreading calipers. Mediolateral kidney widths were 56.94 mm on the right and 58.84 mm on the left. These measurements are within the expected values for an adult of this stature [11]. Both kidneys had two renal arteries, while the R kidney also demonstrated dual renal veins (Figure 1). Kidneys appeared irregular in shape with potential signs of sclerosis. No other gross renal abnormalities were noted following complete uncovering of the renal capsule. 

Internal genitalia

**Figure 2:**
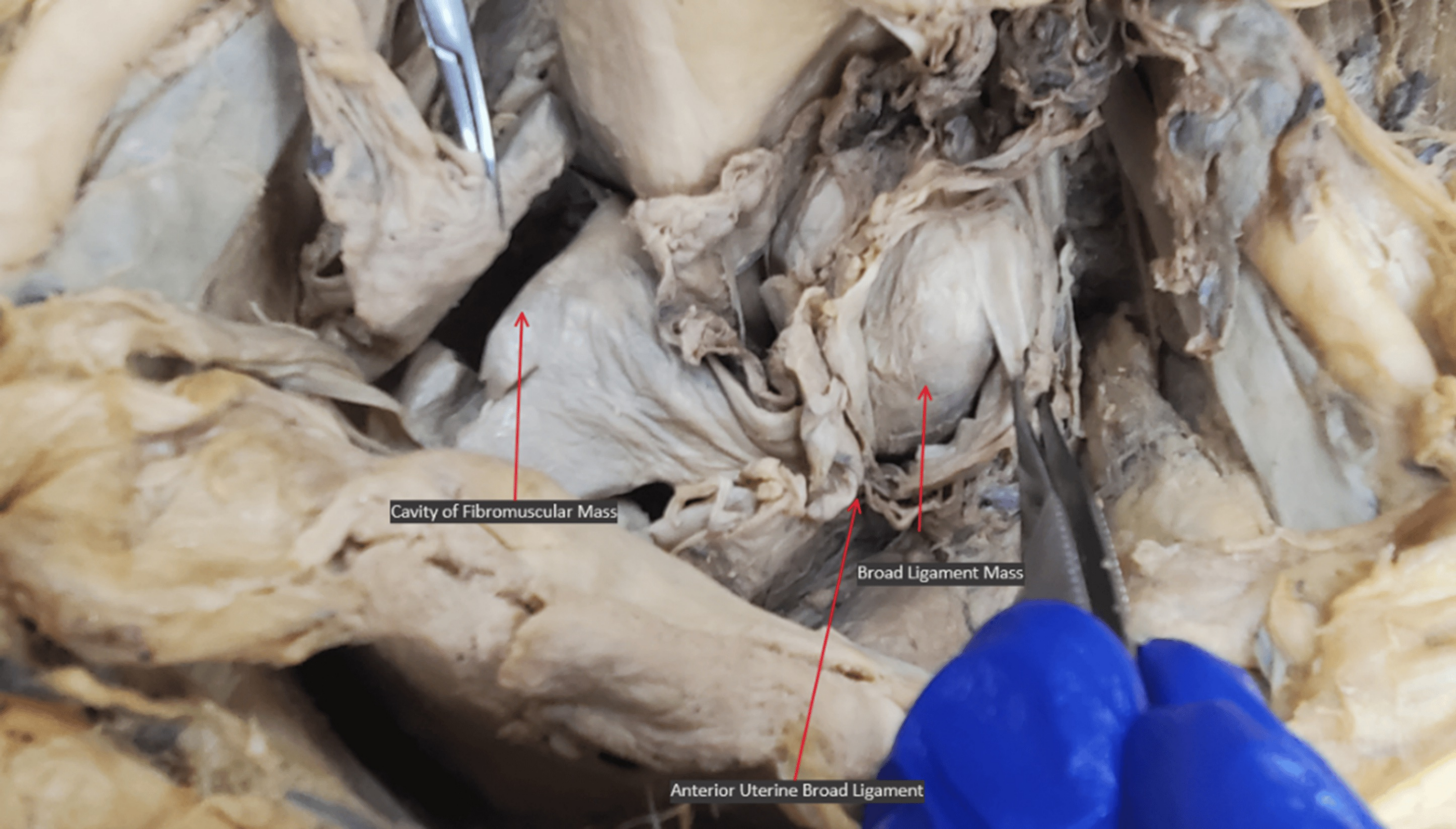
Superior view of the pelvic cavity with a left-side broad ligament mass in situ and right-side fibromuscular mass removed.

**Figure 3:**
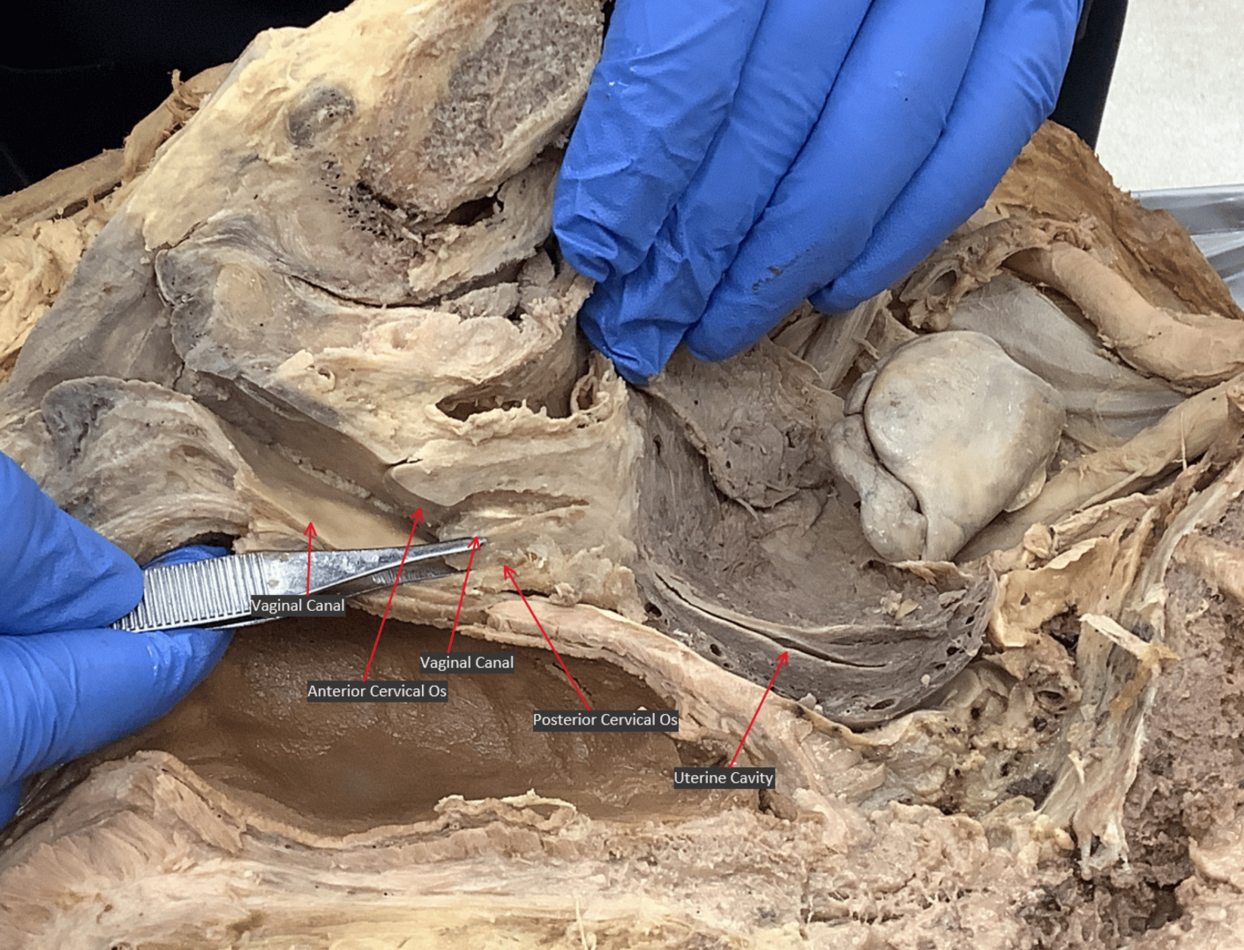
Medial view of the right side of the pelvis after hemisection. Vaginal canal and external os of the cervix are visible, as is the cavity of the uterine body (red arrows).

Two masses were identified inferior to the broad ligament (Figure 2). A first mass made of a fibrous material was present on the left side within the broad ligament and measured 46.05 mm x 26.15 mm x 37.16 mm. A second larger and heavily calcified mass was present within the fibromuscular tissue of the uterus and measured 63.34 mm x 44.18 mm x 55.18 mm. The right and left uterine masses and walls were separated by a medial ligamentous septation. Posterior to the masses sat the uterine cavity, which was approximately 5.7 cm in length. Interspersed within the uterine body were small with <1 cm cavitations. The anterior uterine wall was approximately one-third the density and size of the posterior uterine wall. The tunneling of the external os was about 3 cm in length, and there was no internal os present (Figure 3).

External genitalia

No abnormalities were observed in the external genitalia.

GI tract

The stomach was larger than expected, having thin, muscular walls and diameters at the body and fundus greater than typical based on comparison to other donors (Table 1). The spleen also showed organomegaly [12]. Lastly, the cecum, transverse colon, and rectum were enlarged. External hemorrhoids were also noted.

**Table 1:** Measurements collected from the abdominal organs using spreading calipers.

Organ	Description	Measurement (mm)
Stomach	Wall thickness	0.85
	Diameter at body	103.68
	Diameter at fundus	108.29
Spleen	Length	87.31
	Width	138.84
Cecum	length	143.60
	Diameter	83.56
Transverse colon	Diameter	72.59
Rectum	Superior diameter	44.73
	Inferior diameter	24.51

## Discussion

This report presents gross anatomy associated with an MDA found incidentally in a 78-year-old female cadaver with minimal known medical history. The combination of anatomical variants observed is most consistent with MRKH syndrome with multiple leiomyomas. Several anomalies consistent with MRKH syndrome were noted, including cervical agenesis and uterine hypoplasia with incomplete muscular cavitations. Ovaries and ovarian tubes were fully developed but asymmetrical due to a mass in the left broad ligament. There was no indication of renal agenesis, though renal arteries and veins were duplicated, a somewhat common renal vasculature variant [13]. Enlargement found in much of the gastrointestinal tract and the presence of extensive external hemorrhoids suggest possible chronic problems with digestion and defecation. While other causes cannot be ruled out, such as patient age, diet, immobility, or prior medical conditions, including former CVA or chronic diabetes mellitus type 2, the authors believe that this could be related to complications of leiomyomas and cervical dysgenesis due to anatomic obstruction. No gross skeletal or cranial abnormalities were noted, which can be seen in rare cases of type B MRKH syndrome; however, more in-depth elucidation of associated skeletal and cardiac anomalies, which are commonly implicated with type B MRKH, could not be performed in the timeframe available.

During development, the female reproductive system forms from the Müllerian duct fusing with the urogenital sinus. Failure to do so - as is the case in MRKH syndrome - results in a lack of connection between internal and external genitalia, which has important and often devastating consequences for patients. MDAs are typically diagnosed based on ultrasonography, CT or MRI imaging as well as karyotyping, serum hormone measurements, and celioscopy. The findings of uterine and cervical hypoplasia in this case are consistent with MRKH syndrome with multiple leiomyomas, yet confirmation of this diagnosis would require medical testing that could not be performed. The authors were unable to find any other cadaveric studies of MDA or MRKH, making this an important exploration of the potential anatomical impacts of MDA and possibly MRKH. Although MRKH is rare, up to 1.2% of anatomical females and 25% of people with infertility have MDA's [14,15]. Furthermore, the potential psychological effects this syndrome has on young female patients makes this an important topic for current medical research.

The anatomy observed included a hypoplastic uterine cavity connected to an endocervical canal that was absent or obstructed at the internal os. It is unclear whether this is a congenital cervical anomaly or the result of cervical stenosis [16]. This uncertainty demonstrates the utility of pairing medical histories with donor patients in anatomical research so that rare congenital conditions, which may go undiagnosed in life, can be differentiated from the results of known medical diagnoses or treatments.

## Conclusions

MRKH is a Müllerian duct developmental anomaly where varying degrees of agenesis are present within the fully developed genitourinary system in female sex patients. Common features include hypoplasia or agenesis of part or all of the uterus and/or cervix. MRKH can present with extragenital complications such as renal, skeletal, hearing, and cardiac anomalies. Depending on the severity and extent of the agenesis, MRKH can have lifelong debilitating effects on a female patient’s health, well-being, and ability to conceive and give birth to a child. Through this case report, the authors aim to highlight this condition, which affects many patients, so that clinicians may be more aware and subsequently provide a higher level of care. 
